# Voriconazole metabolism is associated with the number of skin cancers per patient

**DOI:** 10.1007/s00403-024-03135-5

**Published:** 2024-05-31

**Authors:** Jacqueline I. Ike, Isabelle T. Smith, Dominique Mosley, Christopher Madden, Sarah Grossarth, Briana R. Halle, Adam Lewis, Frank Mentch, Hakon Hakonarson, Lisa Bastarache, Lee Wheless

**Affiliations:** 1 Meharry Medical College, Nashville, TN USA; 2 Vanderbilt University College of Arts and Sciences, Nashville, TN USA; 3 Vanderbilt University School of Medicine, Nashville, TN USA; 4 State University of New York Downstate College of Medicine, Brooklyn, NY USA; 5 Quillen College of Medicine, East Tennessee State University, Johnson City, TN USA; 6 Irvine Department of Dermatology, University of California, Davis, USA; 7 Department of Biomedical Informatics, Vanderbilt University Medical Center, Nashville, TN USA; 8 Children’s Hospital of Philadelphia Center for Applied Genomics, Philadelphia, USA; 9 Tennessee Valley Healthcare System VA Medical Center, Nashville, TN USA; 10 Department of Medicine, Division of Epidemiology, Department of Dermatology, Vanderbilt University Medical Center, 719 Thompson Lane, Suite 26300, Nashville, TN 37215 USA

**Keywords:** Skin cancer, Voriconazole, Organ transplant, Epidemiology, Cohort study, Study approved as exempt non-human subjects research, VUMC IRB #200335

## Abstract

Voriconazole exposure is associated with skin cancer, but it is unknown how the full spectrum of its metabolizer phenotypes impacts this association. We conducted a retrospective cohort study to determine how variation in metabolism of voriconazole as measured by metabolizer status of CYP2C19 is associated with the total number of skin cancers a patient develops and the rate of development of the first skin cancer after treatment. There were 1,739 organ transplant recipients with data on CYP2C19 phenotype. Of these, 134 were exposed to voriconazole. There was a significant difference in the number of skin cancers after transplant based on exposure to voriconazole, metabolizer phenotype, and the interaction of these two (*p* < 0.01 for all three). This increase was driven primarily by number of squamous cell carcinomas among rapid metabolizes with voriconazole exposure (*p* < 0.01 for both). Patients exposed to voriconazole developed skin cancers more rapidly than those without exposure (Fine-Grey hazard ratio 1.78, 95% confidence interval 1.19–2.66). This association was similarly driven by development of SCC (Fine-Grey hazard ratio 1.83, 95% confidence interval 1.14–2.94). Differences in voriconazoles metabolism are associated with an increase in the number of skin cancers developed after transplant, particularly SCC.

## Introduction

Voriconazole is often used for anti-fungal prophylaxis among OTR and is associated with an increased risk for cutaneous squamous cell carcinoma (SCC) development [1–4]. The mechanism for this increased risk is thought to involve the metabolite voriconazole N-oxide (VNO) and its ultraviolet B (UVB)-photoproduct acting together as UV-sensitizers that can generate reactive oxygen species and produce oxidative DNA damage, all of which can contribute to SCC formation [5, 6]. Voriconazole is primarily metabolized into VNO by cytochrome P450 enzyme 2C19 (CYP2C19). Genetic variation in the CYP2C19 gene can lead to varying rates of metabolism for voriconazole and its metabolites [7]. One genotype corresponding to the ultrarapid metabolizer phenotype is associated with more rapid development of skin cancer, although the impact of the full range of metabolizers phenotypes on skin cancer risk is unknown [8]. We conducted this study to assess the hypothesis that patients who metabolize voriconazole more rapidly would develop an increased number of skin cancers.

## Methods

### Cohort and exposure measurement

This project was approved by Vanderbilt University Medical Center’s (VUMC) institutional review board with a non-human subjects’ designation, IRB# 200335, and followed the Strengthening the Reporting of Observational Studies in Epidemiology guidelines [9]. The transplant cohort from which subjects were drawn has been previously described [10]. Briefly, data from Vanderbilt’s electronic health record (EHR)-based de-identified research database, the Synthetic Derivative (SD), and its associated biobank, BioVU was used to identify organ transplant recipients with existing genetic data [11]. To ensure more homogeneous baseline skin cancer risks, we restricted the population to those of predominantly Western European genetic ancestry. We included both solid organ transplant recipients and allogenic stem cell transplant recipients in the cohort to increase power as they were all chronically immunosuppressed and had large numbers of patients exposed to voriconazole. The primary exposures were CYP2C19 metabolizer phenotypes and history of exposure to voriconazole. Stargazer was used to determine star alleles for metabolizer phenotype in the CYP2C19 gene [12, 13]. Phenotypes were determined according to the Clinical Pharmacogenetics Implementation Consortium (CPIC) guidelines [7]. “Rapid” and “ultrarapid” metabolizers were combined into a single “rapid” group, and “poor” and “intermediate” metabolizers were combined into “poor”. Exposure to voriconazole was assessed by searching patient medication lists in the SD for “voriconazole” or “Vfend.” The dates of medication exposure and the first skin cancer after exposure were recorded. For unexposed patients, date of transplant was considered time zero [10].

### Outcome definition

The primary outcome was number and type of skin cancers per patient after transplantation. These variables were collected through manual review of all patient records including pathology reports, clinic notes, procedure notes, and International Classification of Disease (ICD) codes. As VUMC has a high volume of external referrals for Mohs surgery, the date of treatment was used for each since the date of biopsy was often not readily available [14]. For skin cancers treated by non-surgical means (e.g. topical imiquimod or 5-fluorouracil), the date of biopsy was used. Skin cancers that were documented only as “a history of skin cancer” were not included. All efforts were made to type cancers with non-specific ICD codes (e.g. ICD-9 173 “Other and unspecified malignant neoplasm of the skin”), however this could not be done in all cases when pathology reports were not available. Where pathology records and clinical notes were not available, we required inclusion of both a Current Procedural Terminology (CPT) code for the treatment of and the ICD code for the diagnosis of a skin cancer on the same day [14]. Lesions that were called dysplastic nevi were excluded, as well intraepithelial neoplasia without specific diagnosis of malignancy. Basosquamous skin cancers were considered BCC according to the historical diagnosis of ‘BCC with squamous differentiation.’ SCC and SCC in situ were considered separately and together. Due to low numbers, these were grouped for the final analysis. Only the original primary tumor was counted when skin cancers recurred, and excisions of in transit or cutaneous metastases were excluded.

### Statistical analyses

We measured the interaction between metabolizer status and voriconazole exposure with the number of skin cancers using a Poisson regression. Differences between groups were compared using t-tests and Chi Squared tests for continuous and categorical variables, respectively. Fine and Gray’s competing hazards model was used to estimate the relative effects of the covariates on the subdistribution hazard of skin cancer and the competing hazard of death and to estimate hazard ratios (HR) and 95% confidence intervals (CI) using the cmprsk and riskRegression packages in R [15]. Covariates included in the models were age at transplant and a history of exposure to cyclosporine or azathioprine as these two medications have been shown to convey high risks for skin cancer development [16–18]. Exploratory analyses further examined exposure to calcineurin inhibitors cyclosporine and oral tacrolimus, both of which can contribute to skin cancer risk. The proportional hazards assumption was assessed using Schoenfeld residuals plotted against failure time for cause-specific hazards. All analyses were conducted using R v4.0.2 between 9/23/23 and 10/31/23. A two-sided p value < 0.05 was considered statistically significant.

## Results

There were 1,739 OTR with metabolizer phenotypes for CYP2C19 (Table 1). Of these, 134 recipients were treated with voriconazole. There were 683 (39.3%) normal metabolizers, 573 (32.9%) rapid, and 483 (27.8%) poor. Patients with voriconazole exposure tended to be transplanted at a similar age (50.8 years vs. 51.9, *p* = 0.36) and have similar numbers of skin cancers after transplant (1.26 vs. 0.8, *p* = 0.26), but have fewer years of follow-up than those without voriconazole exposure (6.0 vs. 8.4 *p* < 0.01). More patients with voriconazole exposure also had exposure to either cyclosporine or azathioprine (49.3% vs. 37.2%, *p* < 0.01). There was a significant interaction between the effects of voriconazole exposure and metabolizer status on the number of skin cancers, and both metabolizer status and voriconazle exposure were associated with the number of skin cancers per individual (*p* < 0.01 for all three). Rapid metabolizers with voriconazole exposure had the greatest mean number of skin cancers, which was largely driven by development of SCC (Table 2).

**Table 1:** Characteristics of All Patients with Metabolizer Data Included in the Study

Characteristic	Patients not exposed to voriconazole (*n* = 1605)	Patients exposed to voriconazole (*n* = 134)
*Sex*		
Male	993 (61.9%)	82 (61.2%)
Female	612 (38.1%)	52 (38.8%)
*Metabolizer Status*		
Rapid	528 (32.9%)	45 (33.6%)
Normal	637 (39.7%)	46 (34.3%)
Poor	440 (27.4%)	43 (32.1%)
Exposure to cyclosporine or azathioprine	597 (37.2%)	66 (49.3%)
Exposure to calcineurin inhibitors	1365 (85.0%)	103 (76.9%)
Age at Transplant, mean, (SD)	51.9 (12.6)	50.8 (12.6)
Years of follow-up, mean, (SD)	8.4 (6.1)	6.0 (4.7)
Any post-transplant skin cancer	245 (15.3%)	29 (21.6%)
*Organ Type*		
Allogenic stem cell	120 (7.5%)	28 (20.9%)
Heart	244 (15.2%)	10 (7.5%)
Kidney	659 (41.1%)	23 (17.2%)
Lung	66 (4.1%)	66 (49.3%)
Liver	516 (32.1%)	7 (5.2%)

**Table 2:** Mean Number of Skin Cancers per Metabolizer Phenotype and Voriconazole Exposure

Voriconazole exposure	Metabolizer phenotype	Mean number of skin cancers (SD)	Mean number of SCC (SD)	Mean number of BCC (SD)	Mean number of unspecified skin cancers (SD)
Unexposed	Rapid (*n* = 528)	0.68 (3.23)	0.471 (2.63)	0.12 (0.64)	0.10 (0.57)
	Normal (*n* = 637)	0.69 (2.52)	0.41 (1.80)	0.18 (1.05)	0.09 (0.55)
	Poor (*n* = 440)	1.11 (4.57)	0.67 (3.04)	0.26 (1.66)	0.18 (0.96)
Exposed	Rapid (*n* = 45)	2.20 (7.35)	1.27 (4.19)	0.47 (1.62)	0.44 (2.83)
	Normal (*n* = 46)	0.91 (2.69)	0.46 (1.36)	0.15 (0.89)	0.30 (1.01)
	Poor (*n* = 43)	0.65 (1.48)	0.40 (1.28)	0.09 (0.48)	0.16 (0.62)

### Time to skin cancer

**Fig. 1:**
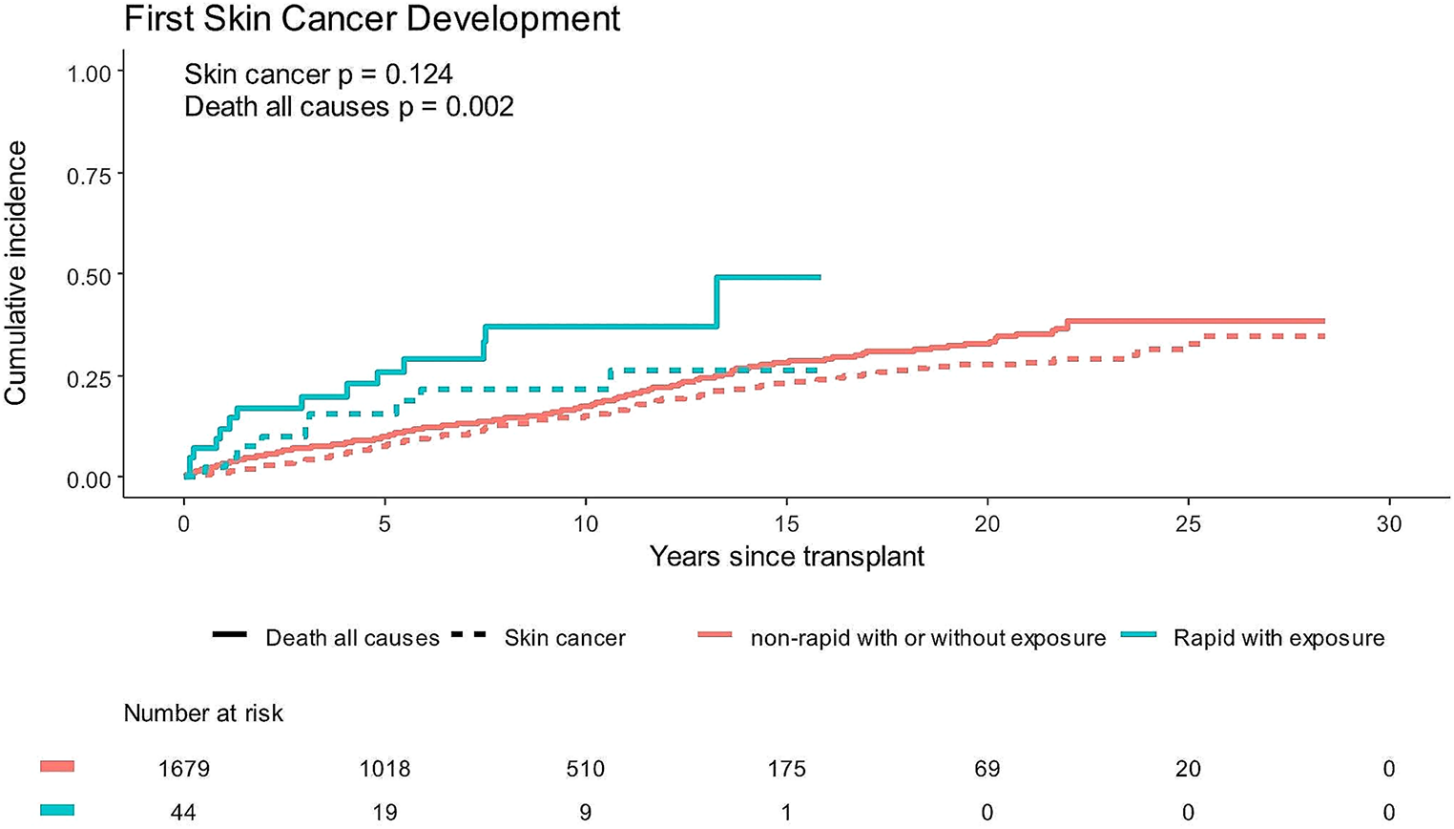
Cumulative incidence of skin cancer or death from all causes in patients who are CYP2C19 rapid metabolizers with voriconazole exposure compared to all other patients

After adjusting for age at transplant and exspoure to cyclosporine or azathioprine, voriconazole exposure was associated with skin cancer risk (HR 1.78, 95% CI 1.19–2.66, *p* < 0.01) (Table 3). This association was driven primarily by development of SCC (HR 1.83, 95% CI 1.14–2.94). Metabolizer phenotype was not associated with time to first skin cancer after adjustment for age and immunosuppressants (*p* = 0.58), but the interaction between voriconazole and metabolizer phenotype was (interaction *p* = 0.03). Because rapid metabolizers with voriconazole exposure had the greatest number of skin cancers on average, we compared these to all phenotypes grouped together. The rapid metabolizer with voriconazole exposure group had an elevated rate of skin cancer development compared to the rest, although had limited power (unadjusted HR 1.66, 95% CI 0.86–3.23, *n* = 10 skin cancers, *p* = 0.13) (Fig. 1). In this model, death was significantly increased among rapid metabolizers with voriconazole exposure. No models showed deviation from the proportional hazards assumpation (*p* > 0.05 for each). In models adjusting for calcinuerin inhibtor exposure, the point estimates were minimally impacted (Table 3). Additional analyses were not pursued due to lack of power.

## Discussion

In this EHR-based retrospective cohort study, we observed a significant increase in skin cancers based on voriconazole metabolizer status among patients exposed to voriconazole, as well as a 78% increased rate for the development of a first skin cancer. There was a significant interaction between metabolizer status and voriconazole exposure contributing to both time to skin cancer and number of skin cancers per patient. Each of these associations were driven by development of SCC in particular.

Our findings support previous work that has shown an increased odds of skin cancer among those treated with voriconazole, and that variation in CYP2C19 impacts this association [3, 4, 8]. Specifically, our study broadens the scope from individual genotypes to established functional metabolizer phenotypes [7, 8]. Our results show not only an increased rate at which patients develop a first skin cancer, but also that OTR develop a greater number of skin cancers per patient after treatment with voriconazole. Our findings are consistent with others who have shown a 73% increased hazard of squamous cell carcinoma in a model that did not account for the competing risk of death [19]. Currently, the Clinical Pharmacogenetics Implementation Consortium (CPIC) guidelines recommend that rapid and ultrarapid metabolizers using an antifungal that is not metabolized by CYP2C19 when antifungal treatment is necessary [7]. Rapid and ultrarapid metabolizer status can often be inferred clinically by patients who have trough levels consistently below predicted ones. As we observed in our study, data from the UK Biobank showed that rapid and ultrarapid metabolizers for CYP2C19 make up roughly one in every three individuals across nearly all genetic populations, indicating that clinical genotyping is very likely to impact treatment decisions and risk profiles [20]. Other agents such as posaconazole and isavuconazole exist, though voriconazole remains the first-line agent for invasive aspergillosis and its prophylaxis [21]. Proton pump inhibitors and glucocorticosteroids can increase or decrease voriconazole levels, respectively [22]. Co-administration of these medications among rapid voriconazole metabolizers can lead to larger shifts in drug levels, and place patients at an increased risk of developing side effects. While there is hope that less voriconazole exposure among this high-risk group will lead to decreased skin cancer risks, dermatologists should be aware of the interaction with metabolizer phenotypes when making decisions on follow-up intervals and prophylaxis.

Rapid metabolizers with voriconazole exposure had increased mortality compared to all other groups, and these patients were more likely to die than to develop skin cancer. Confounding by indication likely is a main driver of this differential mortality in that higher risk patients are getting more antifungal prophylaxis, and potentially a greater intensity of immunosuppression. There was no change in the strength of association for skin cancer when we accounted for classes of immunosuppressants used, nor by age at transplant. These results indicate that rather than a survival bias in which patients who live longer develop the outcome at higher rates, our study might actually be seeing an attenuation of the effects of voriconazole due to decreased survival.

We have shown in this cohort that age at transplant is a stronger predictor of skin cancer development and mortality than transplanted organ type, while different immunosuppression regimens are well-known to increase skin cancer risk [17, 18]. After adjusting for these factors, we still observed an association between metabolizer status, voriconazole exposure and time to skin cancer. More rapid metabolism of voriconazole requires higher doses to reach therapeutic levels. While not direct mechanistic evidence, our results could be consistent with a role for VNO in the development of skin cancer. Alternatively, higher voriconazole levels could lead to greater inhibition of CYP3A4 and CYP3A5. These enzymes are involved in the metabolism of cyclosporine and tacrolimus, which contribute to skin cancer risk [23, 24]. Blocking the metabolism of these medications would trigger a decrease in their dosage to maintain proper levels. Lower cumulative exposures could lead to a lower risk of skin cancer. When we accounted for exposure to calcineurin inhibitors, there was no material impact on the overall association, suggesting that any voriconazole effect on CYP3A4 and CYP3A5 inhibition likely plays a minor role. Similarly, both cyclosporine and azathioprine are known to increase skin cancer risk. When we adjusted for these, the strength of association for voriconazole exposure actually increased, while the interaction of voriconazole and metabolizer phenotype showed minimal change (Table 3).

**Table 3:** Fine-Gray survival models for the risk of developing any skin cancer or SCC with the competing hazard of death from all causes

	Unadjusted model HR (95% CI)	Model 1^a^ HR (95% CI)	Model 2^b^ HR (95% CI)
Voriconazole exposure	All skin cancer: 1.69 (1.14–2.5) SCC: 1.72 (1.08–2.75)	All skin cancer: 1.78 (1.19–2.66) SCC: 1.83 (1.14–2.94)	All skin cancer: 1.73 (1.16–2.59) SCC: 1.82 (1.13–2.92)
Metabolizer phenotype	All skin cancer: Poor: 1.05 (0.79–1.39) Rapid: 0.90 (0.68–1.20) SCC: Poor: 1.15 (0.81–1.62) Rapid: 0.82 (0.57–1.17)	All skin cancer: Poor: 1.03 (0.77–1.37) Rapid: 0.92 (0.69–1.23) SCC: Poor: 1.13 (0.80–1.61) Rapid: 0.83 (0.58–1.19)	All skin cancer: Poor: 1.01 (0.76–1.35) Rapid: 0.92 (0.69–1.23) SCC: Poor: 1.14 (0.80–1.62) Rapid: 0.82 (0.57–1.19)
Voriconazole exposure plus rapid metabolizer phenotype	All skin cancer: 1.66 (0.86–3.23) SCC: 1.77 (0.82–3.83)	All skin cancer: 1.58 (0.80–3.10) SCC: 1.62 (0.74–3.54)	All skin cancer: 1.53 (0.78–3.00) SCC: 1.66 (0.76–3.63)

a – adjusted for age at transplant and exposure to cyclosporine or azathioprine

b – adjusted for age at transplant and exposure to calcineurin inhibitors

Our study had a number of limitations. First, this was a retrospective, single-institution study that might not be generalizable to the broader OTR population. Most importantly, we had low numbers of skin cancers and low numbers of patients with voriconazole exposure with limited power to detect weaker associations. Still, each of the skin cancers included in this study was hand-reviewed from the patient chart, providing an extremely high-quality measure for the outcome of skin cancer counts that few studies are able to provide. Next, we had considerable imbalances in organ type between those exposed and unexposed to voriconazole, and we included both solid and liquid organ recipients. We previously have shown that age at transplant is more important than organ type, and so in our regression models we adjusted for age at the time of transplant, which can vary widely based on transplant indication and organ type. There was a large variance in the number of skin cancers patients developed (Table 2). We attempted to control for this finding by using Poisson regression that could capture the zero-inflated distribution of count data. Because fewer than 50% of patients in each group developed any skin cancer, we could not use methods that would be less impacted by outliers in testing the median. There is the potential for immortal time bias as not all patients received voriconazole starting at the time of transplant. This was a small subset, and the interval was at most 2 weeks, during which time none of them were treated for skin cancer. The cumulative exposure of voriconazole is associated with increasing risk of skin cancers [4]. The medication data in our dataset was of too poor quality to estimate cumulative dose, although it stands to reason that rapid metabolizers would need an increased dosage to maintain therapeutic trough levels, so our metabolizer phenotypes likely accounted for some of the variability in drug exposure. Lastly, to improve power we condensed metabolizer phenotypes “Poor” and “Intermediate” into a single category, which led to some loss of information.

## Conclusions

In this single-center study of 1,739 organ transplant recipients, we observed that rapid metabolism of voriconazole is associated with both an increased number of skin cancers per patient, and an increased rate of skin cancer development. These impacts were independent of age at transplant and exposure to cyclosporine, tacrolimus or azathioprine. Low numbers of events limited our power and these results would need to be replicated in a larger sample size before making clinically-actionable recommendations.

## Data Availability

No datasets were generated or analysed during the current study.

## Abbreviations

BCC: Basal cell carcinoma
CI: Confidence interval
CPIC: Clinical Pharmacogenetics Implementation Consortium
CPT: Current procedural terminology
CYP2C19: Cytochrome P450 2C19
EHR: Electronic health record
HR: Hazard ratio
ICD: International classification of disease
OTR: Organ transplant recipients
SCC: Squamous cell carcinoma
SD: Synthetic Derivative
VNO: Voriconazole-N-oxide
VUMC: Vanderbilt University Medical Center
